# MARS and RNAcmap3: The Master Database of All Possible RNA Sequences Integrated with RNAcmap for RNA Homology Search

**DOI:** 10.1093/gpbjnl/qzae018

**Published:** 2024-03-01

**Authors:** Ke Chen, Thomas Litfin, Jaswinder Singh, Jian Zhan, Yaoqi Zhou

**Affiliations:** Institute of Systems and Physical Biology, Shenzhen Bay Laboratory, Shenzhen 518055, China; Peking University Shenzhen Graduate School, Shenzhen 518055, China; University of Science and Technology of China, Hefei 230026, China; Suzhou Institute for Advanced Research, University of Science and Technology of China, Suzhou 215123, China; Institute for Glycomics, Griffith University, Southport, QLD 4222, Australia; Institute of Systems and Physical Biology, Shenzhen Bay Laboratory, Shenzhen 518055, China; Institute of Systems and Physical Biology, Shenzhen Bay Laboratory, Shenzhen 518055, China; Institute of Systems and Physical Biology, Shenzhen Bay Laboratory, Shenzhen 518055, China; Peking University Shenzhen Graduate School, Shenzhen 518055, China; Institute for Glycomics, Griffith University, Southport, QLD 4222, Australia

**Keywords:** RNA sequence database, Homology search, Secondary structure, MARS, RNAcmap3

## Abstract

Recent success of AlphaFold2 in protein structure prediction relied heavily on co-evolutionary information derived from homologous protein sequences found in the huge, integrated database of protein sequences (Big Fantastic Database). In contrast, the existing nucleotide databases were not consolidated to facilitate wider and deeper homology search. Here, we built a comprehensive database by incorporating the non-coding RNA (ncRNA) sequences from RNAcentral, the transcriptome assembly and metagenome assembly from metagenomics RAST (MG-RAST), the genomic sequences from Genome Warehouse (GWH), and the genomic sequences from MGnify, in addition to the nucleotide (nt) database and its subsets in National Center of Biotechnology Information (NCBI). The resulting Master database of All possible RNA sequences (MARS) is 20-fold larger than NCBI’s nt database or 60-fold larger than RNAcentral. The new dataset along with a new split–search strategy allows a substantial improvement in homology search over existing state-of-the-art techniques. It also yields more accurate and more sensitive multiple sequence alignments (MSAs) than manually curated MSAs from Rfam for the majority of structured RNAs mapped to Rfam. The results indicate that MARS coupled with the fully automatic homology search tool RNAcmap will be useful for improved structural and functional inference of ncRNAs and RNA language models based on MSAs. MARS is accessible at https://ngdc.cncb.ac.cn/omix/release/OMIX003037, and RNAcmap3 is accessible at http://zhouyq-lab.szbl.ac.cn/download/.

## Introduction

There are two major categories of RNAs: those coding for proteins [messenger RNAs (mRNAs)] and those not [non-coding RNAs (ncRNAs)]. The first ncRNA discovered was transfer RNA (tRNA) in 1958 [1]. Since then, new types of ncRNAs were constantly uncovered once every a few years [2]. These ncRNAs can have a length ranging from ∼ 20 nt in microRNAs (miRNAs) [3] to more than 100 kb for long ncRNAs (lncRNAs) like antisense *Igf2r* RNA (*Air*) [4]. These RNAs can perform functions at the sequence level by simple complementary base-pairing in the case of miRNAs [3], at the secondary structural level in the case of protein-directed RNA switches [5], and at the tertiary structural level in the cases of tRNAs, ribosomal RNAs (rRNAs), ribozymes, and riboswitches [6]. The number of distinct ncRNAs likely exceeds that of distinct proteins [7]. This is exemplified by the fact that our human genome dedicates more than 70% to RNA transcripts, compared with a tiny 1.5% coding for proteins [8]. These ncRNAs actively participate in essentially all biological processes and are implicated in more than 1000 diseases [2,9]. Given the increasing importance of annotated and unannotated RNAs in biology (coding and non-coding), a comprehensive sequence database for all RNAs is necessary.

The most comprehensive database for ncRNAs is perhaps RNAcentral [10], which consolidates 56 expert databases and over 30 million sequences as of Jan 2022 (release 20). Another widely used sequence library is nucleotide (nt) database in National Center of Biotechnology Information (NCBI) [11]. Unlike RNAcentral, NCBI’s nt database contains both RNA and DNA sequences. It combines sequences from the databases including GenBank, European Nucleotide Archive (ENA) at the European Molecular Biology Laboratory-European Bioinformatics Institute (EMBL-EBI), and DNA Data Bank of Japan, amounting to 72.9 million sequences as of Aug 2021. However, neither RNAcentral nor NCBI’s nt database is complete for all possible RNA sequences, as many specialized databases and depositories, such as Genome Warehouse (GWH) [12,13] and metagenomics RAST (MG-RAST) [14,15], are not included.

Recently, AlphaFold2 achieved an incredible feat of accurate protein structure prediction for most predicted proteins in the biannual meeting of 14th Critical Assessment of protein Structure Prediction (CASP 14) [16]. This success was in part built on the utilization of homologous sequences to extract evolution and co-evolution information, which contains implicitly the information on sidechain–sidechain distances and backbone/sidechain torsion angles. To secure as many homologous sequences as possible, they utilized the Big Fantastic Database (BFD) covering over 2 billion protein sequences from reference databases, metagenomes, and metatranscriptomes.

Inspired by BFD, we built the Master database of All possible RNA Sequences (MARS). As that in the nt database, we incorporated both RNA sequences and DNA sequences (*i.e.*, genomic sequences). Genomic sequences were included because a large portion of genomic sequences are transcribed into coding RNAs and ncRNAs. Their inclusions allow us to account for all possible (or potential) RNAs.

To illustrate the usefulness of the MARS database, we compared the ability to obtain homologous sequences by using the fully automatic pipeline RNAcmap [17]. In this RNAcmap pipeline, a query sequence is first searched against a database by the Basic Local Alignment Search Tool for Nucleotide (BLAST-N) [18], followed by a covariance model-based search by Infernal [19]. The resulting multiple sequence alignment (MSA) was then evaluated by direct-coupling analysis (DCA) tools such as mfDCA, an algorithm based on the mean-field approximation of DCA [20]. Evolution and co-evolution information obtained from RNAcmap were found useful in improving RNA secondary structure and tertiary base-pair prediction in SPOT-RNA2 [21] as well as distance contact map prediction in SPOT-RNA-2D [22]. In the latest update of RNAcmap (RNAcmap2) [23], an additional search by Infernal was performed on the MSA produced by RNAcmap. A slightly expanded database was also utilized in RNAcmap2 by integrating environment samples (env_nt), transcriptome shotgun assembly (tsa_nt), and nucleotide sequences derived from the Patent Division of GenBank (pat_nt) databases, in addition to NCBI’s nt database. The additional iteration as well as the database expansion was found effective in improving the quality of MSA obtained by examining the accuracy of base-pairs extracted from the MSA using DCA [23]. More recently, an rMSA pipeline was also proposed [24] and found useful in predicting RNA distance and orientation maps by deep learning [25]. It performed five iterative searches against Rfam [26], RNAcentral [10], the nt database [11] by using BLAST-N [18], nhmmer [27], and Infernal [19].

Here, we established MARS database by incorporating the RNAcentral database [10], the transcriptome assembly and metagenome assembly from MG-RAST [14,15], the genomic sequences from GWH [12,13], and the genomic sequences from MGnify [28], in addition to the nt database and its subsets in NCBI. MARS database was about 20-fold and 60-fold larger than the NCBI’s nt database and the RNAcentral database, respectively. We illustrated the usefulness of MARS by employing a data splitting strategy coupled with the homology search tool RNAcmap2. The resulting tool RNAcmap3 increases the median number of effective homologous sequences (N_eff_) by 34.7 folds and the F1-score for base-pair prediction by DCA by 1.4 folds compared with RNAcmap2 for no-hit RNAs (those RNAs lacking homologs according to RNAcmap). RNAcmap3 also yields more accurate MSAs than rMSA as well as manually curated MSAs from Rfam for the majority of structured RNAs mapped to Rfam. MSAs generated by RNAcmap3 have been employed to establish an RNA language model (RNA-MSM), with demonstrated improvement in prediction of RNA secondary and tertiary base-pairs as well as solvent accessible surface area [29].

## Database implementation

### Data collection

The MARS database integrates all available nucleotide sequences, ranging from well-annotated individual nucleotide sequences to poorly understood metagenomics assemblies. Specifically, the data source of MARS includes the nt database and its subsets (env_nt, tsa_nt, and pat_nt) in NCBI [11], the ncRNA sequences from RNAcentral [10], the transcriptome assembly and metagenome assembly from MG-RAST [14,15], the genomic sequences from GWH [12,13], and the genomic sequences from MGnify [28].

The nt, env_nt, tsa_nt, and pat_nt databases were downloaded from ftp://ftp.ncbi.nlm.nih.gov/blast/db on August 27, 2021. The RNAcentral database was obtained from https://ftp.ebi.ac.uk/pub/databases/RNAcentral/current_release/sequences on August 17, 2021. The MG-RAST database was established by collecting assembled transcriptomic and metagenomic sequences from https://www.mg-rast.org on October 7, 2021. The GWH database was downloaded from ftp://download.big.ac.cn/gwh on August 21, 2021. The MGnify database was downloaded from ftp://ftp.ebi.ac.uk/pub/databases/metagenomics/mgnify_genomes on December 21, 2021.

### Data processing

The NCBI databases were downloaded in NCBI-BLAST format. The corresponding fasta files were extracted by blastdbcmd from the BLAST+ 2.12.0 package [30]. The RNAcentral database was downloaded as a zipped fasta file and was used as is after inflation. The MG-RAST, GWH, and MGnify databases were downloaded as individual sequences for assemblies. Sequences from the three sources were first merged according to their data source, resulting three bulk fasta files. The fasta files of MG-RAST and GWH were further formatted as follows: (1) sequences longer than 1000 Mb (which are usually chromosomes) were deleted; (2) all sequences were transferred to DNA alphabet; (3) all gaps, dashes, and non-AT(U)CG characters in sequences were substituted with character “N”. After processing, all eight databases (nt, env_nt, tsa_nt pat_nt, RNAcentral, MG-RAST, GWH, and MGnify) were available as eight bulk fasta files.

The aforementioned databases were concatenated in fasta format, resulting a raw total size of 1744 Gb. SeqKit [31] was then employed to remove 100% duplicated sequences. The final database was versioned as MARS 1.0. It was released in fasta format, and comprised of 1,727,789,860 nucleotide sequences with 1,592,396,862,523 bases in total and file size reaching 1571 Gb, compared with 72.9 million sequences in nt database and 27 million sequences in RNAcentral. The detailed statistical information for all incorporated databases is listed in Table S1. The expansion of MARS from the nt and RNAcentral databases was mainly contributed by the inclusion of the metagenomic and metatranscriptomic sequences from MG-RAST (22.5 folds of nt without redundancy reduction) and NCBI’s env_nt (1.3 folds of nt).

## Benchmark method

### Application to RNA homology search by RNAcmap

**Figure 1:**
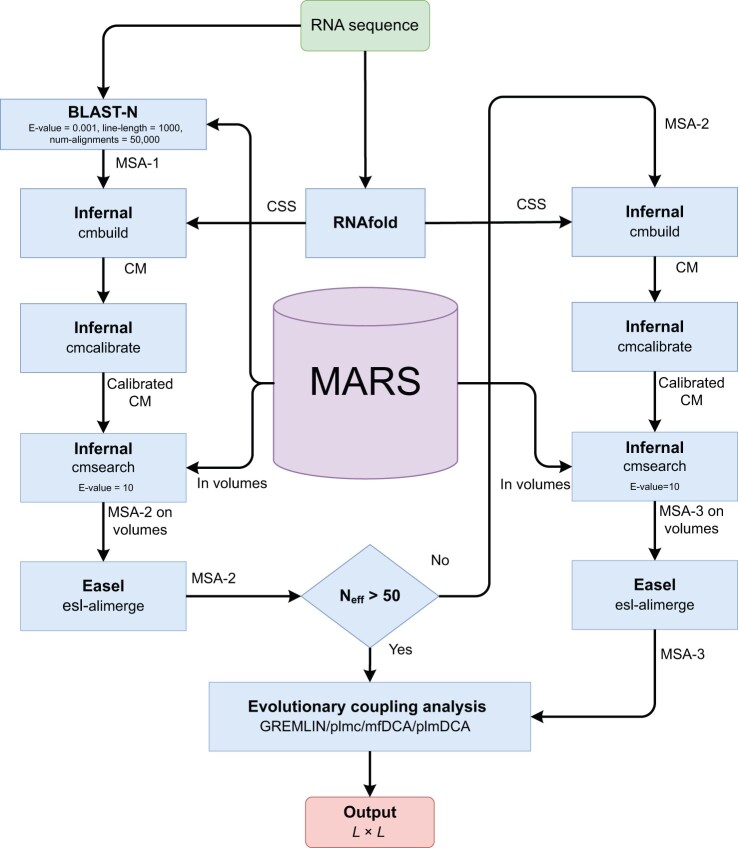
The schematic diagram of the RNAcmap3 pipeline For a given RNA sequence, NCBI BLAST-N is launched first to generate an initial MSA (MSA-1) from MARS, and CSS of the sequence is obtained from a single-sequence secondary structure predictor such as RNAfold. A CM is built with Infernal cmbuild from MSA-1 and CSS, and calibrated with Infernal cmcalibrate. Subsequently, Infernal cmsearch is launched with the calibrated CM to generate a set of secondary MSAs (MSA-2 on volumes) by searching each volume of MARS. This set of MSAs is merged into a complete MSA (MSA-2) with Easel esl-alimerge. MSA-2 is evaluated by its N_eff_ to determine whether it is adequate to launch evolutionary coupling analysis: if N_eff_ > 50, MSA-2 is subjected to the evolutionary coupling analysis tools for the final output; otherwise, another round of Infernal search is launched with MSA-2 as the input MSA for Infernal cmbuild, and the MSA generated in this round (MSA-3) is used for evolutionary coupling analysis. *L* refers to the length of the input RNA sequence. NCBI, National Center of Biotechnology Information; BLAST-N, the Basic Local Alignment Search Tool for Nucleotide; MARS, the Master database of All possible RNA Sequences; N_eff_, the number of effective homologous sequences; MSA, multiple sequence alignment; CSS, consensus secondary structure; CM, covariance model.

The usefulness of the new database was illustrated by homology search. Here, we adapted the three-iteration framework of RNAcmap2 for homology search [23] with a major change on how the databases were searched (**Figure 1**). As one large file for the sequence dataset is inefficient to handle, it was split into 149 volumes with a fixed size of 10 Gb. Independent cmsearch processes in Infernal [19] were evoked on these individual volumes, producing individual MSAs on the volumes. The individual MSAs were then merged into a MSA on the full database with esl-alimerge, a miniapp from Easel toolkit shipped with Infernal. This split–search strategy significantly improves the depth of resulting MSAs. To distinguish this change from RNAcmap2 in relation to the database search, we labeled the current search as RNAcmap3 against the MARS dataset for comparison with the previous RNAcmap results. A simple introduction to the usage of RNAcmap3 with examples is provided in File S1.

### Benchmark for comparing homology searches

We employed the same benchmark datasets that were employed for comparing RNAcmap2 with RNAcmap [23]. Briefly, non-redundant RNA structures (80% cutoff by cd-hit-est [32]) were obtained from Protein Data Bank (PDB) [33]. Their sequences were searched against the nt database by RNAcmap. The MSAs thus generated were evaluated by N_eff_. By its definition, N_eff_ is frequently employed to quantify the homology abundance of RNAs. According to the N_eff_ values calculated by RNAcmap, the RNAs with non-redundant structures were divided into four sets: no-hit (N_eff_ = 0), low N_eff_ (1 ≤ N_eff_ < 10), medium N_eff_ (10 ≤ N_eff_ < 50), and high N_eff_ (N_eff_ ≥ 50), which were composed of 21, 83, 31, and 110 RNAs, respectively. Here, we focused on no-hit, low N_eff_, and medium N_eff_ sets only, because co-variational DCA of the MSAs for the high N_eff_ set has achieved highly accurate prediction of base-pairs by RNAcmap. More homologous sequences by RNAcmap2 or RNAcmap3 can no longer increase evolutionary or co-evolutionary information for those with high N_eff_ by RNAcmap. The aforementioned 135 PDB structures (no-hit, low N_eff_, and medium N_eff_ structures) were further mapped onto Rfam and non-Rfam families by simply searching PDB RNA sequences on the Rfam website (https://rfam.xfam.org). This led to 30 different Rfam families along with 105 sequences that were not mapped to any Rfam families. The MSAs and base-pair predictions from Rfam were compared to those from RNAcmap2 as done by Singh et al. [23] and to those from RNAcmap3 developed here.

The MSAs produced by RNAcmap3 were evaluated by assessing the accuracy of the secondary structure predicted by co-variational analysis of the MSAs, as in RNAcmap2 [23], according to sensitivity [SN = TP/(TP + FN)], precision [PR = TP/(TP + FP)], and F1-score [F1-score = 2 × PR × SN/(PR + SN)] for non-local base-pairs (|*i* − *j*| > 3). Here, TP, FN, and FP are true positives, false negatives, and false positives, respectively. The F1-score, PR, and SN were calculated with the top *L*/3 predictions as predicted truth, where *L* refers to the length of the input RNA sequence. As in RNAcmap2, the co-variational analysis of MSAs was done by DCA predictors (GREMLIN [34], mfDCA [20], plmc [35,36], and plmDCA [37]). These predictors produced mostly similar results among each other and the best performance was given by mfDCA. Thus, we reported the results from mfDCA in subsequent analysis, and provided the results from other methods in supplementary materials including Table S2 and Figure S1 for GREMLIN, Table S3 and Figure S2 for plmc, and Table S4 and Figure S3 for plmDCA.

rMSA is a recently reported pipeline for RNA homology search [24] that searches against nt and RNAcentral databases. Here, the versions of these two databases for rMSA were the same as those used in RNAcmap3. The rMSA program was downloaded from https://github.com/pylelab/rMSA. In all searches, rMSA ran with the default parameters.

The RNAcmap2 results presented here were obtained on the same version of NCBI databases as those used in MARS.

## Comparison with existing databases and homology detection methods

### Performance comparison on RNA homology search

**Figure 2:**
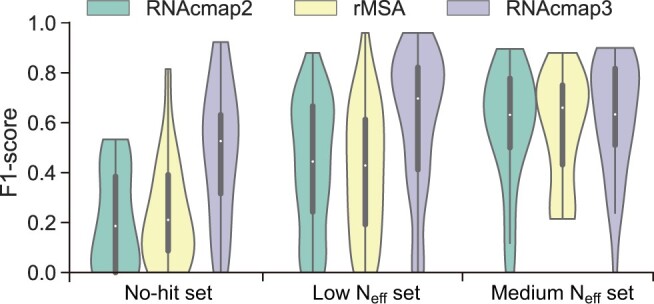
Violin plot of F1-scores predicted by mfDCA using MSAs generated by RNAcmap2, rMSA, and RNAcmap3 The density estimation is computed for no-hit set (21 RNAs), low N_eff_ set (83 RNAs), and medium N_eff_ set (31 RNAs), respectively. In the violin plot, the empty circle denotes the median F1-score, the thick vertical bar in the center denotes the interquartile range, and the thin vertical bar shows the range of data points within another 1.5 interquartile range extension from the thick bar ends. The violin plot is cut off at the range of all actual data points. No-hit means N_eff_ = 0, low N_eff_ means 1 ≤ N_eff_ < 10, and medium N_eff_ means 10 ≤ N_eff_ < 50.

**Table 1** compares the MSAs generated by RNAcmap2, rMSA, and RNAcmap3 in terms of median N_eff_ and average F1-score given by mfDCA for the MSAs. The distribution of F1-scores for individual RNAs is shown in **Figure 2**.

**Table 1:** Performance comparison between RNAcmap2, RNAcmap3, and rMSA on benchmark datasets

Dataset	Pipeline	F1-score	PR	SN	Median N_eff_
No-hit set (21 RNAs)	RNAcmap2	0.204	0.218	0.206	3.0
	rMSA	0.226	0.243	0.223	10.0
	RNAcmap3	**0.483**	**0.501**	**0.489**	**107.1**
Low N_eff_ set (83 RNAs)	RNAcmap2	0.426	0.472	0.396	13.5
	rMSA	0.408	0.446	0.383	25.1
	RNAcmap3	**0.611**	**0.667**	**0.574**	**156.5**
Medium N_eff_ set (31 RNAs)	RNAcmap2	0.587	0.658	0.541	86.4
	rMSA	0.576	0.639	0.553	183.9
	RNAcmap3	**0.628**	**0.692**	**0.604**	**307.1**

*Note*: F1-scores were predicted by mfDCA and averaged over RNAs in each set. No-hit means N_eff_ = 0, low N_eff_ means 1 ≤ N_eff_ < 10, and medium N_eff_ means 10 ≤ N_eff_ < 50. SN = TP / (TP + FN), PR = TP / (TP + FP), and F1-score = 2 × PR × SN / (PR + SN). The best value for each metrics is indicated in bold. N_eff_, the number of effective homologous sequences; SN, sensitivity; PR, precision; TP, true positive; FN, false negative; FP, false positive.

RNAcmap2 and rMSA have a comparable performance in all three datasets. Although rMSA produced MSAs with much higher median N_eff_ values than that of RNAcmap2 in two of three datasets (no-hit set, *P* = 0.039; low N_eff_ set, *P* = 0.100; medium N_eff_ set, *P* = 0.011; *t*-test for the means of two independent sets of samples), no significant difference was observed between the average F1-scores generated by RNAcmap2 and rMSA (Table 1). It seems that a higher N_eff_ value (a statistically significant difference between N_eff_ values with a *P* value of 0.006 for three datasets combined) does not necessarily produce a higher MSA quality (a statistically insignificant difference between F1-scores with a *P* value of 0.628 for three datasets combined).

RNAcmap3 outperforms both RNAcmap2 and rMSA in no-hit and low N_eff_ datasets on all performance indicators with comparable performance on the medium N_eff_ set in terms of medium F1-score. RNAcmap3 increased F1-scores over RNAcmap2 in average by 136.8% for no-hit set, 43.4% for low N_eff_ set, and 7.0% for medium N_eff_ set, respectively. RNAcmap3 also increased F1-scores over rMSA in average by 113.7% for no-hit set, 49.8% for low N_eff_ set, and 9.0% for medium N_eff_ set, respectively. The RNAcmap3-generated MSAs had median N_eff_ values much higher than those of rMSA-generated MSAs. RNAcmap3 produced MSAs with median N_eff_ > 100 even for no-hit set. Comparing with RNAcmap2, the higher median N_eff_ values (*P* = $3.74\times 10^{-13}$) for three datasets combined were indeed related to much better MSA qualities as reflected by the F1-scores (*P* = $4.14\times 10^{-7}$). Note that there was a zero F1-score for RNAcmap3 in the medium N_eff_ set (PDB: 1g1x_E) due to the poor performance of RNAfold for providing initial secondary structure, which was employed in homology search. This leads to a smaller median F1-score for RNAcmap3 on medium N_eff_ RNAs, compared with that for rMSA (Figure 2). More discussions can be found below.

Among three RNA datasets, the improvement of RNAcmap3 is most significant for no-hit and low N_eff_ sets. In fact, the performance of RNAcmap3 on no-hit set is better than that of RNAcmap2 on low N_eff_ set. RNAcmap3 in low N_eff_ set also outperforms RNAcmap2 in medium N_eff_ set. This is consistent with the drastically increased N_eff_ values. The performance improvement of RNAcmap3 on medium N_eff_ set is < 10% over RNAcmap2 (or rMSA), because RNAcmap2 also generates MSAs with sufficient N_eff_ values. This is in line with the notion that prediction accuracy by co-variational analysis along with MSA depth has an upper limit. Similar results (Tables S2–S4; Figures S1–S3) were obtained when GREMLIN, plmc, or plmDCA was employed to measure the quality of MSA.

### Performance comparison between RNAcmap3 and Rfam

Rfam clusters RNA sequences into families according to the homology in sequence and secondary structure. When possible, Rfam utilizes experimentally determined secondary structures for homology search and alignment. By comparison, a method like RNAcmap or rMSA employs RNAfold for initial secondary structure prediction. Thus, Rfam is often considered as the gold standard for RNA MSAs, although not all RNAs in Rfam employ experimentally determined secondary structures.

**Figure 3:**
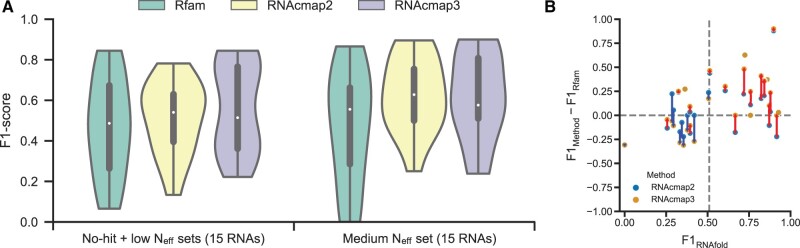
Performance comparison between RNAcmap3 and Rfam **A**. Violin plot of F1-scores predicted by mfDCA using MSAs generated by Rfam, RNAcmap2, and RNAcmap3 for RNAs mapped to Rfam. The density estimation is computed for no-hit set (1 RNA), low N_eff_ set (14 RNAs), and medium N_eff_ set (15 RNAs), respectively. In the violin plot, the empty circle denotes the median F1-score, the thick vertical bar in the center denotes the interquartile range, and the thin vertical bar shows the range of data points within another 1.5 interquartile range extension from the thick bar ends. The violin plot is cut off at the range of all actual data points. No-hit means N_eff_ = 0, low N_eff_ means 1 ≤ N_eff_ < 10, and medium N_eff_ means 10 ≤ N_eff_ < 50. **B**. The difference between the F1-scores given by RNAcmap3/RNAcmap2 (F1_method_) and the F1-scores given by Rfam (F1_Rfam_) as a function of the F1-scores given by RNAfold (F1_RNAfold_). RNAcmap3 and RNAcmap2 results are shown in orange and blue, respectively. The results of RNAcmap3 and RNAcmap2 for same targets are linked with a red line if RNAcmap3 outperforms RNAcmap2, and a blue line if RNAcmap2 outperforms RNAcmap3.

**Figure 3**A shows the F1-scores from mfDCA-predicted base-pairs (top *L*/3) using the MSAs (1 RNA in no-hit set, 14 RNAs in low N_eff_ set, and 15 RNAs in medium N_eff_ set) from Rfam, RNAcmap2, and RNAcmap3, respectively. For medium N_eff_ set, RNAcmap3 generally performed comparable with RNAcmap2 in terms of medium F1-score, except for a few RNAs with poor performance of RNAfold (F1_RNAfold_ < 0.5), including 2DU4_C (Rfam family: RF00005, F1_RNAfold_ = 0.345, F1_RNAcmap2_ = 0.704, F1_RNAcmap3_ = 0.556), 3Q3Z_A (Rfam family: RF01786, F1_RNAfold_ = 0.419, F1_RNAcmap2_ = 0.847, F1_RNAcmap3_ = 0.576), and 6FZ0_A (Rfam family: RF01826, F1_RNAfold_ = 0.286, F1_RNAcmap2_ = 0.524, F1_RNAcmap3_ = 0.238) (Figure S4). This result indicates that for those RNAs with poor RNAfold prediction (F1_RNAfold_ < 0.5), more homologous sequences generated by RNAcmap3 may lead to more false positives and yield poorer performance. For those RNAs with F1_RNAfold_ > 0.5, homologous sequences generated by RNAcmap3 yielded improved F1-scores for most RNAs compared with those by RNAcmap2. For no-hit and low N_eff_ sets, the performance of RNAcmap3 was significantly improved over RNAcmap2 on 9 of 15 Rfam families (Figure S4).

A more detailed comparison for each Rfam family is shown in **Table 2**. In the 30 mapped families, RNAcmap3 outperformed Rfam in 17 families, and Rfam performed better than RNAcmap3 in 10 families, with equal performance on 3 families. On the other hand, RNAcmap3 outperformed RNAcmap2 in 15 families, and RNAcmap2 outperformed RNAcmap3 in 9 families, with equal performance on 6 families. RNAcmap3 was improved over RNAcmap2 when both outperformed Rfam (10 in 18 families). In the 9 families that RNAcmap2 did not perform as well as Rfam, RNAcmap3 improved the performance in 5 families, while failed to do so on the remaining 4 families. On the average, RNAcmap2 performed better on Rfam-mapped RNAs than on non-Rfam RNAs. Interestingly, RNAcmap3 performed even slightly better on non-Rfam RNAs than on Rfam-mapped RNAs. We found that RNAcmap3 was improved substantially over RNAcmap2 on non-Rfam RNAs largely due to large increase of N_eff_ from an average of 49.0 to an average of 248.7. When the number of homologous sequences is sufficiently large, it seems to reinforce the initial secondary structure provided by RNAfold. Indeed, there is a strong correlation between F1-scores generated by RNAfold and those by mfDCA from RNAcmap3 MSAs (see Figure S4 for Rfam-mapped RNAs and Figure S5 for non-Rfam RNAs). Thus, the slightly better performance of RNAcmap3 on non-Rfam RNAs is due to slightly better RNAfold performance on non-Rfam RNAs than on Rfam-mapped RNAs.

**Table 2:** Performance comparison between Rfam, RNAcmap2, RNAcmap3, and RNAfold on Rfam-mapped and non-Rfam RNAs

Rfam family	RNA type	No. of RNAs	PDB chain	F1-score			
				Rfam (N_eff_)	RNAcmap2 (N_eff)_	RNAcmap3 (N_eff_)	RNAfold
RF00005	tRNA	1	2DU4_C	**0.778 (3037.1)**	0.704 (59.0)	0.556 (712.4)	0.345
RF00008	Hammerhead ribozyme	1	2QUS_A	0.604 (171.0)	**0.792 (149.2)**	**0.792 (1340.9)**	0.824
RF00026	U6 spliceosomal	1	4N0T_B	0.000 (2412.8)	0.439 (227.9)	**0.465 (158.2)**	0.513
RF00100	7SK	1	5LYU_A	**0.844 (1832.6)**	0.622 (45.9)	**0.844 (192.9)**	0.916
RF00102	VA	1	6OL3_C	0.409 (29.7)	**0.782 (65.4)**	**0.782 (404.3)**	0.860
RF00164	Coronavirus 3′ stem-loop II-like motif	1	1XJR_A	0.412 (4.0)	0.588 (21.7)	**0.824 (204.1)**	0.824
RF00228	Hepatitis A virus internal ribosome entry site	1	6MWN_A	0.090 (1)	**0.716 (633.2)**	**0.716 (249.0)**	0.725
RF00390	UPSK	1	6MJ0_A	0.066 (1)	0.306 (26.8)	**0.316 (135.8)**	0.324
RF00442	Guanidine-I riboswitch	1	5T83_A	**0.556 (152.7)**	0.250 (300.1)	0.247 (169.4)	0.000
RF00458	Cripavirus internal ribosome entry site	1	2IL9_A	0.391 (14.0)	**0.628 (93.5)**	0.565 (128.3)	0.506
RF00505	RydC	1	4V2S_Q	0.270 (4)	0.526 (65.9)	**0.571 (1271.1)**	0.606
RF01344	CRISPR RNA direct repeat element	1	6JDV_B	0.000 (6.8)	0.880 (132.5)	**0.900 (432.3)**	0.898
RF01415	Flavivirus 3′ UTR stem loop IV	1	4PQV_A	**0.267 (4.9)**	0.133 (9.9)	0.222 (206.9)	0.256
RF01689	AdoCbl variant	1	4FRN_A	**0.613 (60.5)**	0.427 (12.3)	0.507 (107.1)	0.394
RF01704	Downstream peptide RNA	1	6QN3_A	**0.733 (52.3)**	**0.733 (38.7)**	**0.733 (156.0)**	0.759
RF01725	SAM-I/IV variant riboswitch	1	4L81_A	0.500 (169.8)	0.611 (118.2)	**0.750 (574.0)**	0.761
RF01734	Fluoride riboswitch	1	4ENA_A	**0.800 (242.2)**	0.629 (52.4)	0.514 (106.0)	0.333
RF01750	ZMP/ZTP riboswitch	1	4XWF_A	**0.622 (102.4)**	0.444 (262.8)	**0.622 (490.1)**	0.667
RF01763	Guanidine-III riboswitch	1	5O69_A	**0.513 (5.1)**	**0.513 (4.1)**	0.359 (112.8)	0.378
RF01786	Cyclic di-GMP-II riboswitch	1	3Q3Z_A	**0.847 (372.1)**	**0.847 (332.8)**	0.576 (650.6)	0.419
RF01826	SAM-V riboswitch	1	6FZ0_A	0.300 (2.9)	**0.524 (24.1)**	0.238 (2251.0)	0.286
RF01852	Selenocysteine transfer	1	3ADB_C	0.866 (298.8)	**0.896 (56.9)**	**0.896 (1002.5)**	0.928
RF02519	ToxI antitoxin	1	4ATO_G	0.091 (1.2)	**0.364 (15.3)**	**0.364 (156.5)**	0.364
RF02553	Y RNA-like	1	6CU1_A	**0.698 (84.2)**	0.476 (330.6)	0.387 (381.7)	0.355
RF02678	Hatchet ribozyme	1	6JQ5_A	0.222 (3.2)	0.444 (9.0)	**0.704 (84.1)**	0.720
RF02679	Pistol ribozyme	1	6UFJ_A	0.486 (43.3)	**0.541 (47.6)**	0.378 (849.0)	0.294
RF02680	PreQ1-III riboswitch	1	4RZD_A	0.261 (2.8)	0.294 (11.9)	**0.353 (110.7)**	0.393
RF02683	NiCo riboswitch	1	4RUM_A	0.627 (118.2)	0.667 (61.7)	**0.866 (625.4)**	0.879
RF02796	Pab160	1	3LWO_D	0.462 (3.3)	0.667 (13.5)	**0.821 (592.8)**	0.842
RF03013	nadA	1	6TFE_A	0.737 (28.8)	0.632 (14.0)	**0.842 (190.3)**	0.872
Mean	–	–	–	0.469 (308.8)	0.569 (107.9)	**0.590 (468.2)**	0.575
Non-Rfam	–	105	–	–	0.389 (49.0)	**0.596 (248.7)**	0.634

*Note*: F1-scores were predicted by mfDCA. The best value for each metrics is indicated in bold. PDB, Protein Data Bank.

One big difference between Rfam and RNAcmap is that Rfam relies on known secondary structures whereas RNAcmap employs secondary structures predicted by RNAfold. Table 2 and Figure 3B illustrates the dependence of RNAcmap performance on RNAfold. In particular, the improvement of RNAcmap2 or RNAcmap3 over Rfam F1-score was positively correlated with the F1-score given by RNAfold [Pearson’s correlation coefficient (PCC) = 0.599 and *P =* 4.76 × 10^−4^ for RNAcmap3; PCC = 0.358 and *P =* 0.052 for RNAcmap2]. If RNAfold predictions have a F1-score greater than 0.51, RNAcmap3 always performs equally or better than RNAcmap2 and Rfam. We noted that the performance of RNAcmap2 or RNAcmap3 was positively correlated with the performance of RNAfold. For RNAcmap3, the overall PCC between co-variational-derived and RNAfold-predicted secondary structures was 0.964, compared with 0.470 for RNAcmap2. This highlights that more homologous sequences found by RNAcmap3 reinforce the mapping to the seed secondary structures given by RNAfold.

To confirm the dependence of sequences found by RNAcmap3 on the seed secondary structures, we also examined the use of another energy-based method RNAstructure [38] as well as the deep learning technique SPOT-RNA [39]. For SPOT-RNA, we excluded the RNAs in the benchmarking set overlapping with those in the SPOT-RNA training set. As shown in Figures S6 (RNAstructure) and S7 (SPOT-RNA), as in RNAfold, strong correlations (PCC = 0.922 for RNAstructure and PCC = 0.860 for SPOT-RNA) between F1-scores of seed secondary structures and F1-scores of co-variational analysis of sequences obtained from RNAcmap3 based on the seed secondary structures were observed. This result confirms that locating more homologous sequences reinforces the seed secondary structure. It should be noted that improved accuracy of seed secondary structures allows improved co-variation signals, as shown in Tables S5 and S6.

One interesting question is whether RNAcmap2 or RNAcmap3 can locate remote homologs. To illustrate this, we compared median sequence identities to a query sequence given by RNAcmap3 to those given by RNAcmap2. As shown in Figure S8, sequence identities for RNAcmap2 and RNAcmap3 both ranged from 40% to 80%. However, RNAcmap3 often can obtain more RNAs with low sequence identity than RNAcmap2, suggesting that the use of the MARS database allows the discovery of more remote homologs.

## Discussion

In this study, we established a comprehensive database of nucleotide sequences, MARS, by including the nt, env_nt, tsa_nt, and pat_nt datasets from NCBI, the ncRNA sequences from RNAcentral, the transcriptome assembly and metagenome assembly from MG-RAST, the genomic sequences from GWH, and the genomic sequences from MGnify. MARS is more than 20 times larger than the commonly used nt database in the number of sequences. Using a split–search strategy for the MARS database allows RNAcmap3 to gain a deeper MSA and yield better co-evolution coupling than RNAcmap2 and rMSA. Moreover, despite using RNAfold as the initial secondary structure for homology inference, RNAcmap3 can achieve more accurate inference of the secondary structures from MSAs than from Rfam MSAs. RNAcmap3 is expected to be useful for improving RNA homology search.

One issue of MARS is the huge size of the sequence datasets with 1.5 Tb for its first version. This huge size makes the homology search very slow, despite of the strategy of data splitting for parallel processing. A typical search for a 100-nt sequence would take 4 h on 24 central processing units (CPUs). Longer sequences of more than 1000 nt are prohibitively slow. One expects that the sequence database will continue to expand exponentially given the low cost of high-throughput sequencing. Unfortunately, not all datasets contained in the MARS database can be updated fully automatically. For example, an ftp access to the MGnify database with a script frequently suffers from broken connections. One must rely on human intervention to complete the process.

For RNAcmap3, one limitation is that one must use a predicted secondary structure as the initial guess for homology search. As an illustration, we mainly employed RNAfold. We found that the performance of the method is heavily dependent on how accurate is the initial secondary structure prediction (Figure 3B, Figures S4–S7). This problem can be addressed with improved prediction of secondary structure, for example, by deep learning techniques (*e.g.*, SPOT-RNA [39], MXfold2 [40], and UFold [41]). However, there is a risk of overtraining for some of these deep learning techniques, which would make some methods to perform poorly for unseen RNA families [42]. Thus, caution must be exercised when using these deep learning techniques. Despite the limitation for the dependence of the seed secondary structure, the MSAs generated by RNAcmap3 from the MARS database have been used to generate an unsupervised MSA-based RNA language model (RNA-MSM) [29]. The language model improves the prediction of RNA solvent accessibility and secondary and tertiary base-pairs over RNAsnap2 [43] and SPOT-RNA2 [21], respectively, both of which also employ evolution information from RNAcmap.

## Supplementary Material

qzae018_Supplementary_Data

## Data Availability

MARS v1.0 can be accessible at https://ngdc.cncb.ac.cn/omix/release/OMIX003037.

## Code availability

RNAcmap3 can be accessible at http://zhouyq-lab.szbl.ac.cn/download/.

## CRediT author statement

**Ke Chen:** Methodology, Software, Validation, Investigation, Data curation, Writing – original draft, Writing – review & editing, Visualization. **Thomas Litfin:** Conceptualization, Methodology, Writing – review & editing. **Jaswinder Singh:** Methodology, Software, Writing – review & editing. **Jian Zhan:** Conceptualization, Writing – review & editing, Supervision. **Yaoqi Zhou:** Conceptualization, Resources, Writing – original draft, Writing – review & editing, Supervision, Project administration, Funding acquisition. All authors have read and approved the final manuscript.
