# Supplementary material for: MARS and RNAcmap3: The Master Database of All Possible RNA Sequences Integrated with RNAcmap for RNA Homology Search
Source: Genomics Proteomics Bioinformatics. 2024 Mar 1;22(1):qzae018. doi: 10.1093/gpbjnl/qzae018 (PMC12053375; doi:10.1093/gpbjnl/qzae018)
Supplement: qzae018_Supplementary_Data [file qzae018_supplementary_data.zip › File S1.docx]

**File S1 Two examples of MARS usage coupled with RNAcmap3**

RNAcmap3 can be installed according to the instructions in README.md file shipped with the package. The Master database of All possible RNA Sequences (MARS) should be deployed in both NCBI-BLAST format and fasta format. Assume that one installed the program to $program_dir and placed MARS fasta files in $fasta_db_dir and MARS BLAST files in $blast_db_dir with the database name MARS. A homology search can be launched with the following command: $program_dir/run_rnacmap3.sh -i $inputfile -d $dca -b “$blast_db_dir/MARS” -c “$fasta_db_dir” -n $np, where $inputfile points to the location of input RNA sequence file, $dca specifies the direct-coupling analysis (DCA) predictor that one would like to use (currently mfDCA, GREMLIN, plmc, and plmDCA are available), $blast_db_dir/MARS specifies the location of MARS BLAST files, $fasta_db_dir specifies the directory containing MARS fasta files, and $np specifies the number of processes [usually, central processing units (CPU) cores] that one would like to employ for the search.

## Example 1: 1XJR_A

Chain A of Protein Data Bank (PDB): 1XJR [1] is a stem-loop II-like motif belonging to Rfam family RF00164.

The sequence in fasta format is:

>1xjr_A

GGAGUUCACCGAGGCCACGCGGAGUACGAUCGAGGGUACAGUGAAUU

Let’s assume that the fasta file is placed in $workdir as 1xjr_A.fasta. RNAcmap3 can be called by one command like: $program_dir/run_rnacmap3.sh -i $workdir/1xjr_A.fasta -d mfdca -b “$blast_db_dir/MARS” -c “$fasta_db_dir” -n 8. Output files will be written to directory $workdir/1xjr_A_features, and a stream of runtime info will be printed.

Once MSA-2 search completed, GREMLIN can be employed to evaluate the N_eff_ value of the generated MSA file. MSA-3 search will be launched if the calculated N_eff_ value is lower than 50. Depending on whether or not the MSA-3 round search is launched, the result MSA is written to $workdir/1xjr_A_features/1xjr_A.a2m_msa3 (if MSA-3 search is launched) or $workdir/1xjr_A_features/1xjr_A.a2m_msa2 (if MSA-3 search is not launched). Because we obtained a N_eff_ = 204.074 from MSA-2 search ( > 50), MSA-3 search was not launched. The resulted 1xjr_A.a2m_msa2 contains up to first 50,000 hits (3101 hits in this case) aligned to the query sequence with insertion removed for the purpose of DCA analysis. A comprehensive MSA with all hits and insertion info in Stockholm format can be found as 1xjr_A.msa_msa2. However, the Stockholm file can become very large for some long query sequences if a huge number of hits was obtained. The MSA files can be further processed and visualized with common bioinformatics graphical tools that accept fasta or Stockholm files, such as Jalview [2] and MEGA [3]. For our benchmarking purpose, RNAcmap3 also yields one or more DCA analysis files recording a descending list of base-pair formation probability between bases, such as 1xjr_A.dca_gremlin if GREMLIN is selected as the DCA predictor, or 1xjr_A.dca_plmc if plmc is alternatively selected. The DCA results of mfDCA and plmDCA, due to distinct output rules of these DCA tools, could be found in $workdir/MFDCA_output_1xjr_A for mfDCA, and $workdir/PLMDCA_output_1xjr_A for plmDCA.

## Example 2: 6JQ5_A

Chain A of PDB: 6JQ5 [4] is a Hatchet ribozyme belonging to Rfam family RF02678.

The sequence in fasta format is:

>6jq5_A

UUACUGUGAGAAUCAGUAACAAACAUGUGGGGCUUAUAUCUAAUCGAAAGAUUAGUAUUAGUGCAGACGUUAAAACCAUGUC

Assume that the fasta file was placed in $workdir as 6jq5_A.fasta. RNAcmap3 can be called by one command like: $program_dir/run_rnacmap3.sh -i $workdir/6jq5_A.fasta -d mfdca -b “$blast_db_dir/MARS” -c “$fasta_db_dir” -n 8. Output files will be written to directory $workdir/6jq5_A_features, and a stream of runtime info will be printed.

Depending on whether or not MSA-3 round search is launched, the result MSA is written to $workdir/6jq5_A_features/6jq5_A.a2m_msa3 (if MSA-3 search is launched) or $workdir/6jq5_A_features/6jq5_A.a2m_msa2 (if MSA-3 search is not launched). Here we obtained a N_eff_ = 32.637 from MSA-2 search which is below 50. Thus, MSA-3 search is launched. The resulted 6jq5_A.a2m_msa3 contains 311 hits aligned to the query sequence with insertion removed for the purpose of DCA analysis. A comprehensive MSA with insertion info in Stockholm format can be found as 6jq5_A.msa_msa3. The MSA-3 search produced a MSA with N_eff_ = 84.1 and mfDCA F1-score 0.704, compared to the MSA N_eff_ = 9.0 and mfDCA F1-score 0.444 produced by RNAcmap2.

## MSA applications

MARS integrated with RNAcmap3 is a powerful homology detection tool for RNA, especially for those with low homology abundance. The main output as MSA indicates homology among the query sequence and the hit sequences. Homology is a useful clue for single-sequence tasks like template-based structure prediction, and also provides valuable input for data-driven tasks such as deep learning techniques. MSAs generated by RNAcmap3 have been employed to establish an RNA language model (RNA-MSM), with demonstrated improvement in prediction of RNA secondary and tertiary base-pairs as well as solvent accessible surface area [5]

## Data availability

The mentioned input and output files for the two examples, organized as “example 1_1xjr_A.zip” and “example_2 6jq5_A.zip” can be accessed from <http://zhouyq-lab.szbl.ac.cn/download/>.

## References

[1] Robertson MP, Igel H, Baertsch R, Haussler D, Ares M Jr, Scott WG. The structure of a rigorously conserved RNA element within the SARS virus genome. PLoS Biol 2005;3:e5.

[2] Waterhouse AM, Procter JB, Martin DMA, Clamp M, Barton GJ. Jalview Version 2 — a multiple sequence alignment editor and analysis workbench. Bioinformatics 2009;25:1189–91.

[3] Tamura K, Stecher G, Kumar S. MEGA11: Molecular Evolutionary Genetics Analysis Version 11. Mol Biol Evol 2021;38:3022–7.

[4] Zheng L, Falschlunger C, Huang K, Mairhofer E, Yuan S, Wang J, et al. Hatchet ribozyme structure and implications for cleavage mechanism. Proc Natl Acad Sci U S A 2019;116:10783–91.

[5] Zhang Y, Lang M, Jiang J, Gao Z, Xu F, Litfin T, et al. Multiple sequence alignment-based RNA language model and its application to structural inference. Nucleic Acids Res 2023. https://doi.org/10.1093/nar/gkad1031.
