# Supplementary material for: MARS and RNAcmap3: The Master Database of All Possible RNA Sequences Integrated with RNAcmap for RNA Homology Search
Source: Genomics Proteomics Bioinformatics. 2024 Mar 1;22(1):qzae018. doi: 10.1093/gpbjnl/qzae018 (PMC12053375; doi:10.1093/gpbjnl/qzae018)
Supplement: qzae018_Supplementary_Data [file qzae018_supplementary_data.zip › Supplementary material captions.docx]

## Supplementary material

**File S1 Two examples of MARS usage coupled with RNAcmap3**

**Figure S1 Violin plot of F1-score predicted by GREMLIN using MSAs generated by RNAcmap2, rMSA, and RNAcmap3**

The density estimation is computed for No-hit RNAs (21 RNAs), Low N_eff_ RNAs (83 RNAs), and Medium N_eff_ RNAs (31 RNAs), respectively. In the violin plot, the empty circle denotes the median, the thick vertical bar in the center denotes the interquartile range, and the thin vertical bar shows the range of data points that within another 1.5 interquartile range extension from the thick bar ends. The violin plot is cut off at the range of all actual data points. No-hit means N_eff_ = 0, Low N_eff_ means 1 ≤ N_eff_ < 10, and Medium N_eff_ means 10 ≤ N_eff_ < 50.

**Figure S2 Violin plot of F1-score predicted by plmc using MSAs generated by RNAcmap2, rMSA, and RNAcmap3**

The density estimation is computed for No-hit RNAs (21 RNAs), Low N_eff_ RNAs (83 RNAs), and Medium N_eff_ RNAs (31 RNAs), respectively. In the violin plot, the empty circle denotes the median, the thick vertical bar in the center denotes the interquartile range, and the thin vertical bar shows the range of data points that within another 1.5 interquartile range extension from the thick bar ends. The violin plot is cut off at the range of all actual data points. No-hit means N_eff_ = 0, Low N_eff_ means 1 ≤ N_eff_ < 10, and Medium N_eff_ means 10 ≤ N_eff_ < 50.

**Figure S3 Violin plot of F1-score predicted by plmDCA using MSAs generated by RNAcmap2, rMSA, and RNAcmap3**

The density estimation is computed for No-hit RNAs (21 RNAs), Low N_eff_ RNAs (83 RNAs), and Medium N_eff_ RNAs (31 RNAs), respectively. In the violin plot, the empty circle denotes the median, the thick vertical bar in the center denotes the interquartile range, and the thin vertical bar shows the range of data points that within another 1.5 interquartile range extension from the thick bar ends. The violin plot is cut off at the range of all actual data points. No-hit means N_eff_ = 0, Low N_eff_ means 1 ≤ N_eff_ < 10, and Medium N_eff_ means 10 ≤ N_eff_ < 50.

**Figure S4** **The F1-score from DCA of RNAcmap3 (or RNAcmap2) as a function of F1-score from RNAfold for Rfam-mapped RNAs**

The F1-score from DCA of MSAs generated by RNAcmap3 (or RNAcmap2) is plotted as a function of F1-score given by RNAfold for RNAs mapped to Rfam. RNAcmap3 and RNAcmap2 results are shown in orange and blue, respectively. F1-scores for No-hit and Low N_eff_ RNAs are combined because there is only one Rfam-mapped RNA in No-hit set. The results of RNAcmap3 and RNAcmap2 for same targets are linked with a red arrow if RNAcmap3 improves over RNAcmap2, and a blue one, if otherwise. No-hit means N_eff_ = 0, Low N_eff_ means 1 ≤ N_eff_ < 10, and Medium N_eff_ means 10 ≤ N_eff_ < 50. DCA, direct-coupling analysis.

**Figure S5** **The F1-score from DCA of RNAcmap3 (or RNAcmap2) as a function of F1-score from RNAfold for non-Rfam RNAs**

The F1-score from DCA of MSAs generated by RNAcmap3 (or RNAcmap2) is plotted as a function of F1-score given by RNAfold for RNAs not mapped to Rfam. RNAcmap3 and RNAcmap2 results are shown in orange and blue, respectively. The Pearson’s correlation coefficient between F1_RNAfold_ and F1_Method_ are 0.433 (*P =* 0.057, No-hit), 0.503 (*P* = 1.04×10^-5^, Low N_eff_), and 0.535 (*P =* 0.033, Medium N_eff_) for RNAcmap2, and 0.951 (*P =* 1.30×10^-10^, No-hit), 0.983 (*P =* 1.93×10^-51^, Low N_eff_), and 0.949 (*P =* 2.07×10^-8^, Medium N_eff_) for RNAcmap3. The results of RNAcmap3 and RNAcmap2 for same targets are linked with a red arrow if RNAcmap3 improves over RNAcmap2 and a blue one, if otherwise. In Low N_eff_ case the arrows are omitted for clarity. No-hit means N_eff_ = 0, Low N_eff_ means 1 ≤ N_eff_ < 10, and Medium N_eff_ means 10 ≤ N_eff_ < 50.

**Figure S6 The F1-score from DCA of MSA generated by RNAcmap3 as a function of F1-score given by RNAstructure**

The MaxExpect program of RNAstructure is employed**.** The Pearson’s correlation coefficient between F1_RNAstructure_ and F1_RNAcmap3_ are 0.968 (*P =* 6.37×10^-13^, No-hit), 0.920 (*P =* 1.19×10^-34^, Low N_eff_), and 0.884 (*P =* 4.19×10^-11^, Medium N_eff_), respectively. The Pearson’s correlation coefficient over all sequences is 0.922 (*P =* 1.22×10^-56^). No-hit means N_eff_ = 0, Low N_eff_ means 1 ≤ N_eff_ < 10, and Medium N_eff_ means 10 ≤ N_eff_ < 50.

**Figure S7** **The F1-score from DCA of MSA generated by RNAcmap3 as a function of F1-score given by SPOT-RNA**

Sequences from the training and validation sets of SPOT-RNA are excluded. The Pearson’s correlation coefficient between F1_SPOT-RNA_ and F1_RNAcmap3_ are 0.843 (*P =* 1.53×10^-4^, No-hit), 0.867 (*P =* 1.47×10^-18^, Low N_eff_), and 0.870 (*P =* 6.80×10^-8^, Medium N_eff_), respectively. The Pearson’s correlation coefficient over all sequences is 0.860 (*P =* 5.65×10^-29^). No-hit means N_eff_ = 0, Low N_eff_ means 1 ≤ N_eff_ < 10, and Medium N_eff_ means 10 ≤ N_eff_ < 50.

**Figure S8 The median sequence identity of MSAs generated by RNAcmap3 and RNAcmap2**

Each scatter point represents a query from the benchmarking set with its position determined by the median sequence identity of its MSA generated by RNAcmap2 and the median sequence identity of its MSA generated by RNAcmap3. In the calculation of median sequence identity of a MSA, only sequences with a coverage over 50% (aligned to at least 50% of the query sequence) were counted.

**Table S1 Statistical information of the incorporated databases**

**Table S2 Performance comparison among RNAcmap2, RNAcmap3, and rMSA using the GREMLIN predictor**

**Table S3** **Performance comparison among RNAcmap2, RNAcmap3, and rMSA using the plmc predictor**

**Table S4 Performance comparison among RNAcmap2, RNAcmap3, and rMSA using the plmDCA predictor**

**Table S5 Performance of RNAcmap3 with RNAfold, SPOT-RNA, and RNAstructure as SS predictor**

**Table S6 Performance of RNAfold, SPOT-RNA, and RNAstructure predictions of SS**
