# Supplementary material for: MARS and RNAcmap3: The Master Database of All Possible RNA Sequences Integrated with RNAcmap for RNA Homology Search
Source: Genomics Proteomics Bioinformatics. 2024 Mar 1;22(1):qzae018. doi: 10.1093/gpbjnl/qzae018 (PMC12053375; doi:10.1093/gpbjnl/qzae018)
Supplement: qzae018_Supplementary_Data [file qzae018_supplementary_data.zip › Table S1.docx]

**Table S1 Statistical information of the incorporated databases**

| **Database** | **Size in fasta (Gb)** | **Num of sequences** | **Num of bases** | **Average seq length** | **Non-redundant size (Gb)** |
| --- | --- | --- | --- | --- | --- |
| NCBI nt | 490 | 72,867,915 | 510,232,094,340 | 7002.2 | 490 |
| NCBI env_nt | 113 | 93,365,217 | 107,585,220,968 | 1152.3 | 108 |
| NCBI pat_nt | 28 | 45,440,659 | 25,329,731,650 | 557.4 | 13 |
| NCBI tsa_nt | 15 | 16,445,817 | 13,792,908,986 | 838.7 | 14 |
| RNAcentral | 18 | 26,711,206 | 17,005,268,573 | 636.6 | 11 |
| MG-RAST | 658 | 1,640,313,388 | 646,428,503,869 | 394.1 | 536 |
| GWH | 387 | 41,726,820 | 407,576,899,731 | 9767.7 | 365 |
| MGnify | 35 | 16,393,040 | 36,772,262,782 | 2243.2 | 34 |
| Overall | 1744 | 1,953,264,062 | 1,764,722,890,899 | 903.5 | 1571 |

*Note*: For each database, redundancy reduction is performed against all databases listed on top of it. NCBI, National Center of Biotechnology Information; nt, nucleotide database; env_nt, environment samples; tsa_nt, transcriptome shotgun assembly; pat_nt, nucleotide sequences derived from the Patent Division of GenBank databases; MG-RAST, metagenomics RAST; GWH, Genome Warehouse.
