# Supplementary material for: MARS and RNAcmap3: The Master Database of All Possible RNA Sequences Integrated with RNAcmap for RNA Homology Search
Source: Genomics Proteomics Bioinformatics. 2024 Mar 1;22(1):qzae018. doi: 10.1093/gpbjnl/qzae018 (PMC12053375; doi:10.1093/gpbjnl/qzae018)
Supplement: qzae018_Supplementary_Data [file qzae018_supplementary_data.zip › Table S2.docx]

**Table S2 Perfor****mance comparison among RNAcmap2, RNAcmap3, and rMSA using the GREMLIN predictor**

| **Dataset** | **Pipeline** | **F1-score** | **Precision** | **Sensitivity** | **Median N_eff_** |
| --- | --- | --- | --- | --- | --- |
| No-hit RNAs (21 RNAs) | RNAcmap2 | 0.187 | 0.198 | 0.195 | 3.0 |
|  | rMSA | 0.177 | 0.193 | 0.169 | 10.0 |
|  | RNAcmap3 | **0.466** | **0.484** | **0.469** | **107.1** |
| Low N_eff_ RNAs (83 RNAs) | RNAcmap2 | 0.368 | 0.405 | 0.347 | 13.5 |
|  | rMSA | 0.381 | 0.417 | 0.357 | 25.1 |
|  | RNAcmap3 | **0.584** | **0.640** | **0.546** | **156.5** |
| Medium N_eff_ RNAs  (31 RNAs) | RNAcmap2 | 0.518 | 0.583 | 0.476 | 86.4 |
|  | rMSA | 0.490 | 0.547 | 0.471 | 183.9 |
|  | RNAcmap3 | **0.584** | **0.646** | **0.560** | **307.1** |

*Note*: No-hit means N_eff_ = 0, Low N_eff_ means 1 ≤ N_eff_ < 10, and Medium N_eff_ means 10 ≤ N_eff_ < 50. The best value for each metrics is indicated in bold.
