# Supplementary material for: MARS and RNAcmap3: The Master Database of All Possible RNA Sequences Integrated with RNAcmap for RNA Homology Search
Source: Genomics Proteomics Bioinformatics. 2024 Mar 1;22(1):qzae018. doi: 10.1093/gpbjnl/qzae018 (PMC12053375; doi:10.1093/gpbjnl/qzae018)
Supplement: qzae018_Supplementary_Data [file qzae018_supplementary_data.zip › Table S3.docx]

**Table S3 Performance comparison among RNAcmap2, RNAcmap3, and rMSA using the plmc predictor**

| **Dataset** | **Pipeline** | **F1-score** | **Precision** | **Sensitivity** | **Median N_eff_** |
| --- | --- | --- | --- | --- | --- |
| No-hit RNAs (21 RNAs) | RNAcmap2 | 0.218 | 0.235 | 0.215 | 3.0 |
|  | rMSA | 0.238 | 0.248 | 0.243 | 10.0 |
|  | RNAcmap3 | **0.484** | **0.505** | **0.481** | **107.1** |
| Low N_eff_ RNAs (83 RNAs) | RNAcmap2 | 0.394 | 0.434 | 0.368 | 13.5 |
|  | rMSA | 0.433 | 0.475 | 0.405 | 25.1 |
|  | RNAcmap3 | **0.609** | **0.666** | **0.571** | **156.5** |
| Medium N_eff_ RNAs  (31 RNAs) | RNAcmap2 | 0.549 | 0.617 | 0.506 | 86.4 |
|  | rMSA | 0.558 | 0.620 | 0.537 | 183.9 |
|  | RNAcmap3 | **0.612** | **0.673** | **0.591** | **307.1** |

*Note*: No-hit means N_eff_ = 0, Low N_eff_ means 1 ≤ N_eff_ < 10, and Medium N_eff_ means 10 ≤ N_eff_ < 50. The best value for each metrics is indicated in bold.
