# Supplementary material for: MARS and RNAcmap3: The Master Database of All Possible RNA Sequences Integrated with RNAcmap for RNA Homology Search
Source: Genomics Proteomics Bioinformatics. 2024 Mar 1;22(1):qzae018. doi: 10.1093/gpbjnl/qzae018 (PMC12053375; doi:10.1093/gpbjnl/qzae018)
Supplement: qzae018_Supplementary_Data [file qzae018_supplementary_data.zip › Table S5.docx]

**Table S5 Performance of RNAcmap3 with RNAfold, SPOT-RNA, and RNAstructure as SS predictor**

| **Dataset** | **SS predictor** | **F1** | **Precision** | **Sensitivity** | **Median N_eff_** | **No. of RNAs** |
| --- | --- | --- | --- | --- | --- | --- |
| No-hit RNAs | SPOT-RNA | 0.547 | 0.557 | 0.570 | 142.4 | 14 |
|  | RNAstructure | 0.450 | 0.472 | 0.449 | 142.0 | 21 |
|  | RNAfold | 0.488 | 0.507 | 0.490 | 107.1 | 21 |
| Low N_eff_ RNAs | SPOT-RNA | 0.585 | 0.633 | 0.562 | 205.8 | 58 |
|  | RNAstructure | 0.574 | 0.630 | 0.541 | 156.1 | 83 |
|  | RNAfold | 0.607 | 0.663 | 0.569 | 156.5 | 83 |
| Medium N_eff_ RNAs | SPOT-RNA | 0.643 | 0.704 | 0.636 | 254.4 | 23 |
|  | RNAstructure | 0.604 | 0.667 | 0.578 | 335.4 | 31 |
|  | RNAfold | 0.626 | 0.691 | 0.601 | 307.1 | 31 |

*Note*: The results are generated using mfDCA. The MaxExpect program of RNAstructure suite is employed to predict the secondary structures. SS, secondary structure.
