# Supplementary material for: MARS and RNAcmap3: The Master Database of All Possible RNA Sequences Integrated with RNAcmap for RNA Homology Search
Source: Genomics Proteomics Bioinformatics. 2024 Mar 1;22(1):qzae018. doi: 10.1093/gpbjnl/qzae018 (PMC12053375; doi:10.1093/gpbjnl/qzae018)
Supplement: qzae018_Supplementary_Data [file qzae018_supplementary_data.zip › Table S6.docx]

**Table S6 Performance of RNAfold, SPOT-RNA, and RNAstructure predictions of SS**

| **Dataset** | **SS predictor** | **F1** | **Precision** | **Sensitivity** | **No. of RNAs** |
| --- | --- | --- | --- | --- | --- |
| No-hit RNAs | SPOT-RNA | 0.647 | 0.770 | 0.592 | 14 |
|  | RNAstructure | 0.501 | 0.587 | 0.451 | 21 |
|  | RNAfold | 0.533 | 0.604 | 0.489 | 21 |
| Low N_eff_ RNAs | SPOT-RNA | 0.674 | 0.820 | 0.602 | 58 |
|  | RNAstructure | 0.648 | 0.761 | 0.579 | 83 |
|  | RNAfold | 0.646 | 0.740 | 0.588 | 83 |
| Medium N_eff_ RNAs | SPOT-RNA | 0.708 | 0.852 | 0.644 | 23 |
|  | RNAstructure | 0.624 | 0.733 | 0.578 | 31 |
|  | RNAfold | 0.611 | 0.702 | 0.575 | 31 |

*Note*: The MaxExpect program of RNAstructure suite is employed to predict SS.
