# Supplementary figures and images for: MARS and RNAcmap3: The Master Database of All Possible RNA Sequences Integrated with RNAcmap for RNA Homology Search
Source: Genomics Proteomics Bioinformatics. 2024 Mar 1;22(1):qzae018. doi: 10.1093/gpbjnl/qzae018 (PMC12053375; doi:10.1093/gpbjnl/qzae018)

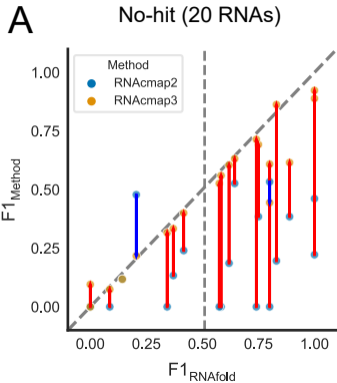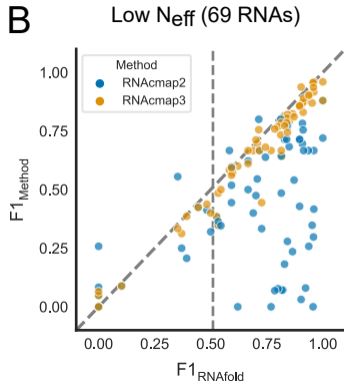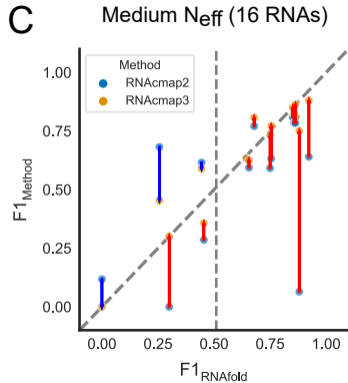

Supplement: qzae018_Supplementary_Data [file qzae018_supplementary_data.zip › Figure S5.pdf]

**A**

No-hit (21 RNAs)

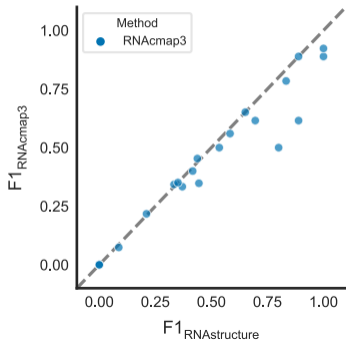**B**Low  $N_{\text{eff}}$  (83 RNAs)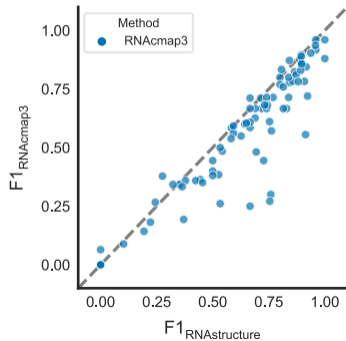**C**Medium  $N_{\text{eff}}$  (31 RNAs)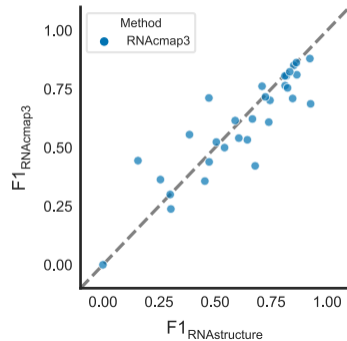

Supplement: qzae018_Supplementary_Data [file qzae018_supplementary_data.zip › Figure S6.pdf]

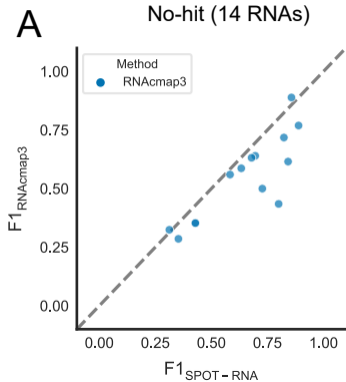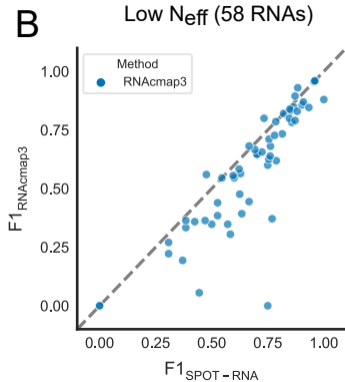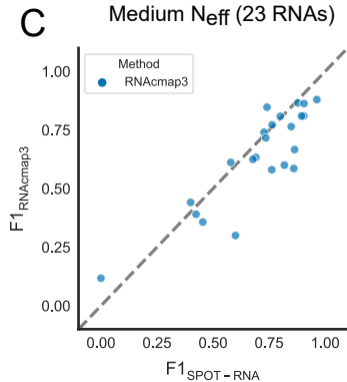

Supplement: qzae018_Supplementary_Data [file qzae018_supplementary_data.zip › Figure S7.pdf]

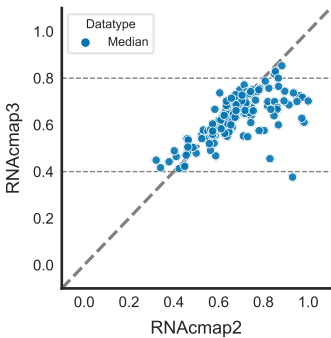

Supplement: qzae018_Supplementary_Data [file qzae018_supplementary_data.zip › Figure S8.pdf]

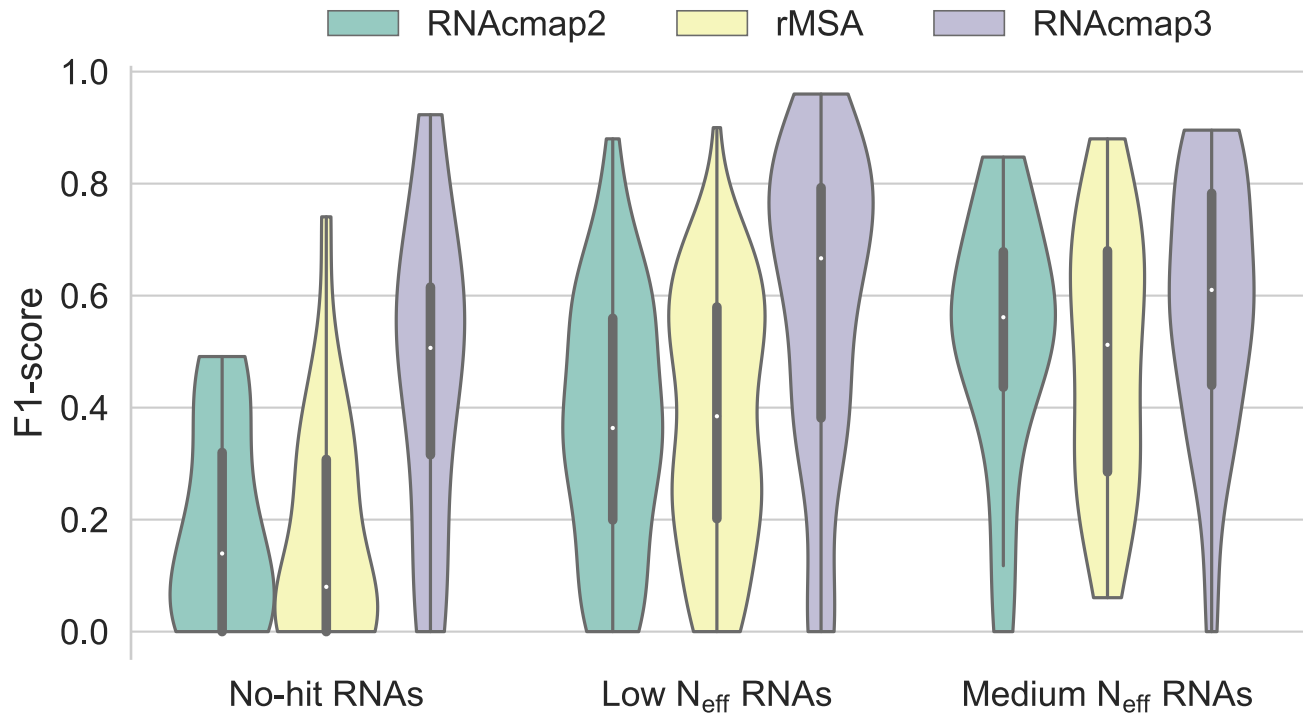

Supplement: qzae018_Supplementary_Data [file qzae018_supplementary_data.zip › Figure S1.pdf]

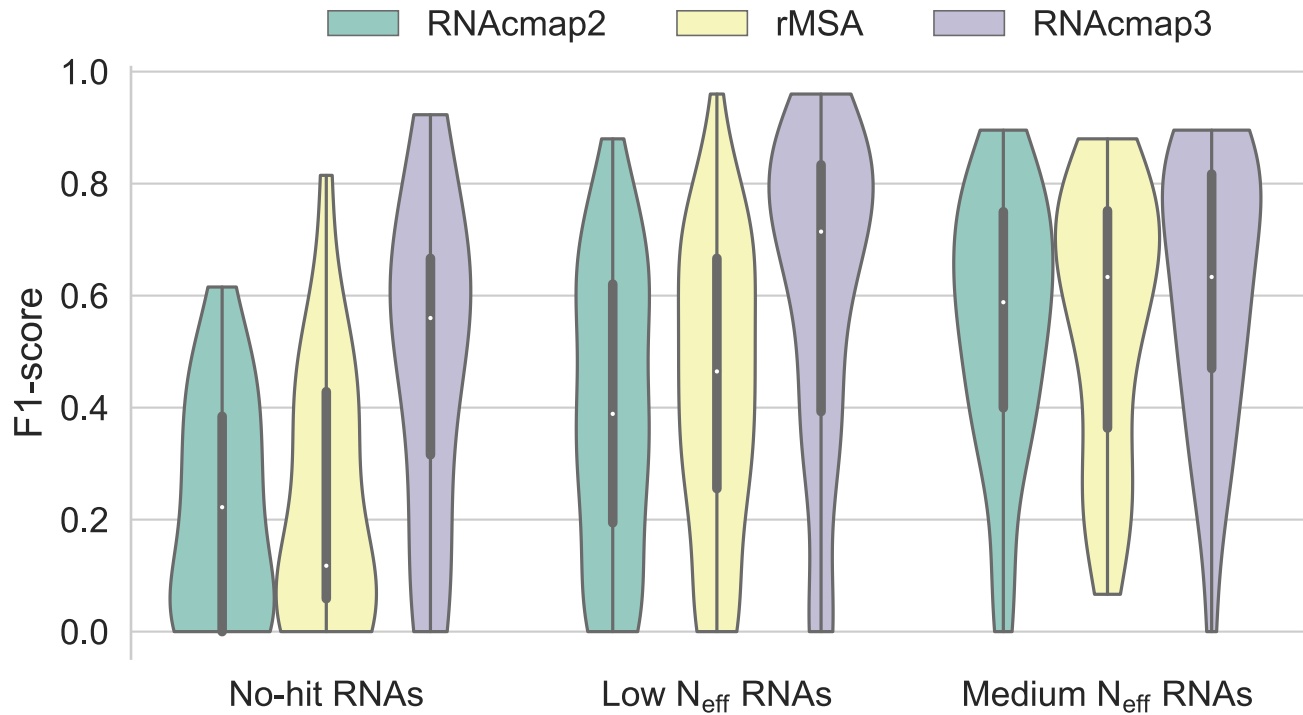

Supplement: qzae018_Supplementary_Data [file qzae018_supplementary_data.zip › Figure S2.pdf]

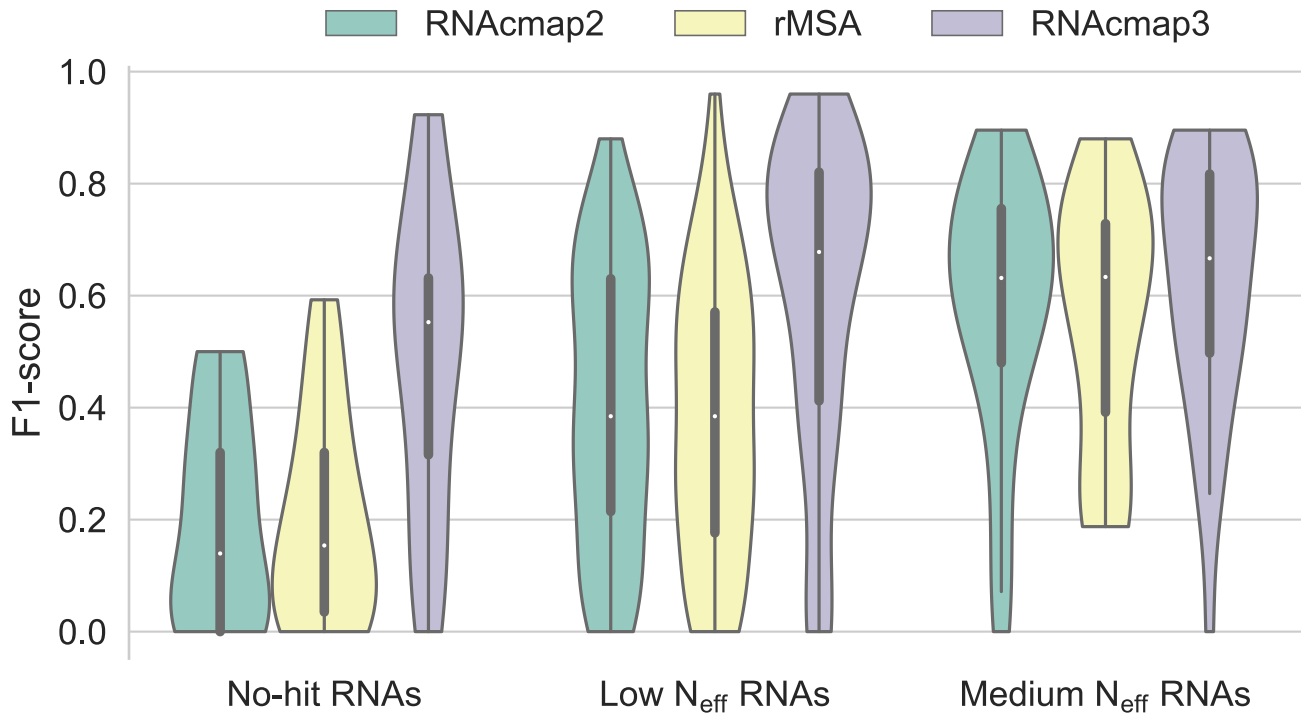

Supplement: qzae018_Supplementary_Data [file qzae018_supplementary_data.zip › Figure S3.pdf]

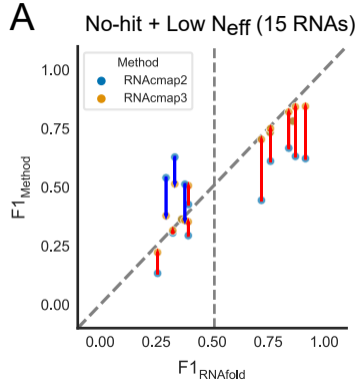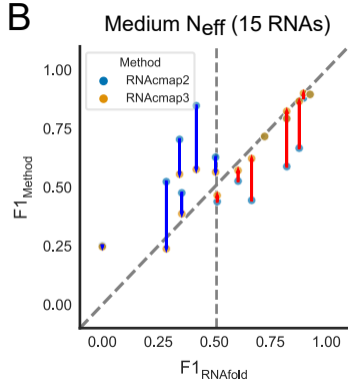

Supplement: qzae018_Supplementary_Data [file qzae018_supplementary_data.zip › Figure S4.pdf]
